# Alexithymia and intolerance of uncertainty predict somatic symptoms in autistic and non-autistic adults

**DOI:** 10.1177/13623613221109717

**Published:** 2022-07-15

**Authors:** Fionnuala Larkin, Brianna Ralston, Sophie Jayne Dinsdale, Sakura Kimura, Marianna Emma Hayiou-Thomas

**Affiliations:** 1 University of York, UK; 2 University College Cork, Ireland

**Keywords:** alexithymia, autism, interoception, intolerance of uncertainty, somatic symptoms

## Abstract

Autistic people experience heightened rates of physical health problems but may also experience elevated levels of somatic symptoms (e.g. pain, headache, gastrointestinal symptoms) due to psychological factors which are common in autism. This online study sought to compare rates of somatic symptoms (Patient Health Questionnaire-15) in older adolescents and adults who were autistic (*n* = 51), non-autistic (*n* = 119) and who suspected they were autistic (*n* = 32), while controlling for health conditions. We investigated psychological risk factors that may predispose individuals to experience somatic symptoms, including alexithymia (General Alexithymia Factor Score), interoception (Body Awareness Questionnaire) and intolerance of uncertainty (Intolerance of Uncertainty Scale). Diagnosed autistic individuals had higher rates of alexithymia and intolerance of uncertainty. We also found higher somatic symptoms in diagnosed autistic individuals, controlling for mental and physical health. However, hierarchical regression showed that somatic symptoms were predicted by physical and mental health conditions, female gender, alexithymia, and intolerance of uncertainty, regardless of autism status. The results suggest that autistic and non-autistic individuals experience more bodily discomfort in part due to gender, physical and mental health, alexithymia, and difficulty tolerating uncertainty. Implications for mental and physical health care in autism are discussed.

**Lay abstract:**

Autistic people have more physical health problems than non-autistic people. We were interested in whether autistic people experience more discomfort in their bodies than non-autistic people and whether certain psychological traits contribute to that. A survey was completed online by older adolescents and adults, 51 of whom were autistic, 32 of whom thought they might be autistic but were not diagnosed and 119 who were not autistic. They completed measures of somatic symptoms (daily experience of pain, discomfort, dizziness), alexithymia (difficulty identifying and expressing feelings), interoception (how much people are aware of their bodies) and intolerance of uncertainty (how people handle doubt or uncertainty), and reported any physical or mental health conditions. We found that the autistic participants had more physical and mental health conditions than the non-autistic participants, but even when we took account of this, they experienced higher levels of somatic symptoms. We looked at which psychological factors influenced levels of somatic symptoms across the whole sample, and found that alexithymia, intolerance of uncertainty, having physical health problems, being female and the number of mental health conditions predicted somatic symptoms, while interoception and autism diagnosis did not. The findings suggest that people may be more likely to experience physical discomfort if they are female, and have difficulty identifying and expressing feeling and difficulty tolerating doubt. As these psychological factors are more prominent in autism, we think this is important for physical and mental health providers to know about, so that these psychological factors can be considered when assessing and treating autistic people.

Autism spectrum conditions (ASCs)^1^ are characterised by difficulties in social communication and reciprocity as well as rigid, repetitive patterns of behaviour, often along with sensory sensitivity (American Psychiatric Association, 2013). Autistic individuals have been found to experience higher levels of medical issues than non-autistic people (Cashin et al., 2018), with many conditions found to be more prevalent in autistic adults compared to non-autistic adults (Fortuna et al., 2016; Matson & Goldin, 2013; Stewart et al., 2020). Biological reasons for higher rates of physical health problems in ASC are unclear, and may link to genetic predisposition, maternal inflammatory conditions, gut microbiota and sensory hypersensitivity (Alabaf et al., 2019; Pan et al., 2020; Penzol et al., 2019). The burden of additional illnesses in ASCs can be considerable, lowering quality of life and well-being (Coales et al., 2019; Ten Hoopen et al., 2020).

Psychological factors are known to influence the experience of physical health problems or the somatic symptoms which comprise these problems. Somatic symptoms are ‘perceived abnormalities of bodily structure or function that the individual finds bothersome or concerning’ (Sharpe, 2013, p. 320). When a medical cause cannot be identified, these can be described as ‘medically unexplained’ (Looper & Kirmayer, 2002). However, maintaining a dichotomy between physical and psychological causes of somatic symptoms is no longer in favour, since the burden of somatic symptoms, whether there is a physical cause or not, is closely linked with functional impairment and need for resources (Sharpe, 2013). Individuals with somatic symptoms may experience physical symptoms for years, which may include frequent headaches, back pain and gastrointestinal (GI) problems (Trimble, 2004), and can comprise functional somatic syndromes such as irritable bowel syndrome (IBS), chronic fatigue and fibromyalgia/chronic pain (Petersen et al., 2020).

Various psychological theories have attempted to explain individuals’ experience of somatic symptoms, from psychoanalytical formulations of conversion disorder (Freud, 1962), to a focus on personality traits of neuroticism and introversion as explanatory factors in functional somatic syndromes (Matthews et al., 2009). Cognitive-behavioural theories have focused on the ways in which anxiety about physical symptoms maintains attention on these sensations (Salkowskis et al., 2003). Across these diverse theoretical perspectives is a unifying focus on somatic symptoms as manifestations or correlates of emotional distress. Health and illness involve more than the physical fitness of the body: emotional factors are involved in how people perceive and interpret physical sensations, how they communicate their experience and how they seek help (Porcelli & Taylor, 2018; Sharpe, 2013). Thus, the experience of somatic symptoms likely involves attentional, cognitive, emotional, behavioural and interpersonal processes.

As such, the question arises as to whether autistic individuals experience higher levels of somatic symptoms, irrespective of the presence or absence of diagnosed physical health conditions. One study in the Netherlands found that autistic adults (*n* = 172) reported significantly higher levels of somatisation on the Symptom Checklist–90–Revised (SCL-90-R) compared to a comparison group of non-autistic adults (*n* = 172) (Lever & Geurts, 2016). Similarly, a study investigating associated disorders prevalent in autistic adults (*n* = 62) found that 10% had Somatic Symptom Disorder (Okamoto et al., 2017). A study of adult women who were either autistic or had ADHD (attention deficit hyperactivity disorder) found that over 75% experienced chronic pain (Asztély et al., 2019). A recent twin study suggested that GI symptoms in autism were likely to stem from behavioural and emotional factors rather than being autism specific (Pan et al., 2020). Some research has reported reduced pain sensitivity in autism using experimental methods (Yasuda et al., 2016); however, a recent experimental study found that autistic adults undergoing a heat pain task, compared to non-autistic adults, rated their pain as higher, and had higher levels of pain anxiety and fear (Failla et al., 2020). This emphasises the need to explore cognitive factors related to pain and other somatic symptoms.

Thus, while there are many recent studies detailing increased prevalence of psychiatric conditions among autistic children and adults (Colvert et al., 2022; Lai et al., 2019), there is a paucity of studies focused specifically on somatic symptoms or somatisation in ASCs. Therefore, the current study sought to establish whether autistic people reported themselves to have higher rates of somatic symptoms than non-autistic people, while controlling for diagnosed physical health problems, and to investigate whether psychological features characteristic of ASCs may render people more vulnerable to experiencing somatic symptoms.

## Alexithymia

Alexithymia is a psychological trait that describes difficulty with noticing and labelling emotional states in oneself (Kafetsios & Hess, 2019). Elevated levels of alexithymia have been found in approximately 50% of autistic individuals, with most exhibiting at least some alexithymic traits (Bird & Viding, 2014; Hill et al., 2004; Lombardo et al., 2007). Therefore, the presence of this trait may predispose autistic individuals to experience and respond to physical sensations in altered ways, and indeed recent empirical work has found associations between alexithymia and somatic symptoms in autism (Williams & Gotham, 2021). Alexithymia is thought to be closely linked to physical health and somatic symptoms through multiple pathways (Porcelli & Taylor, 2018). Individuals high in alexithymia can struggle to recognise internal symptoms, therefore resulting in delays seeking medical attention, resulting in poor prognoses (Kojima, 2012). Alternatively, the physical sensations that accompany emotional responses may be misinterpreted as bodily symptoms: studies have demonstrated that patients presenting with chronic pain have greater levels of alexithymia compared to control groups, and that alexithymia is strongly linked with over-reporting of physical symptoms (Kreitler & Niv, 2001; Lumley et al., 2007). It can be assumed, therefore, that those with alexithymia may perceive and respond to internal bodily signals in atypical ways (Kano & Fukudo, 2013).

## Interoception

Interoception is a sense that allows an individual to perceive their body’s internal state, including sensations such as temperature, itch, hunger, thirst, touch and pain (Khalsa et al., 2018; Quattrocki & Friston, 2014). Research has highlighted both impaired and enhanced interoceptive ability within the ASC population (Shah et al., 2016). Autistic adults reported considerably lower body and thirst awareness, relative to neurotypical controls (Fiene & Brownlow, 2015). However, Garfinkel et al. (2016) found that autistic adults showed impaired ability to *detect* bodily signals alongside a subjective *perception* of elevated bodily sensations. Hatfield et al. (2019) argue that interoceptive differences in autism may relate to difficulties with the integration of bodily signals. Interoceptive difficulties have been implicated as an underlying factor in a variety of psychopathology presentations (Khalsa et al., 2018; Murphy et al., 2017), and can underlie hypersensitivity to bodily sensations, and the tendency to over-report somatic symptoms (Barsky et al., 1988; Fairclough & Goodwin, 2007).

## Intolerance of uncertainty

Intolerance of uncertainty (IU) is a dispositional trait that entails the tendency to view uncertainty as threatening, and it is a risk factor for various anxiety disorders (Birrell et al., 2011; Rodgers et al., 2018). Autistic individuals have greater levels of IU compared to their non-autistic counterparts (Boulter et al., 2014; Neil et al., 2016), which may be a risk factor for anxiety (Vasa et al., 2018). The construct of IU may inadvertently contribute to the manifestation and maintenance of panic and anxiety disorders by influencing the interpretation of physical symptoms (Carleton, 2012). For instance, while heart palpitations are not inherently threatening, if the underlying cause of the palpitations is unknown, individual capacity to tolerate this uncertainty without catastrophising becomes critical (Carleton et al., 2013). In addition, IBS has been strongly associated with heightened levels of anxiety, worry and IU (Drews & Hazlett-Stevens, 2008; Gros et al., 2009). Individuals scoring high on IU and anxiety have more doctor visits and present with more gastric complaints than those low in anxiety (Aldao et al., 2010; Bélanger et al., 2005). Therefore, we investigate whether IU predicts self-report of somatic symptoms in autistic and non-autistic individuals.

### The current study

In summary, the current study sought to (a) examine rates of somatic symptoms in autistic adults compared to non-autistic adults and (b) explore whether certain psychological factors common to ASCs increase the risk of experiencing elevated somatic symptoms. Given previous research, we predicted that autistic adults would have higher levels of somatic symptoms than the non-autistic adults, as well as higher levels of the risk factors under investigation: alexithymia, IU and interoceptive sensibility. We hypothesised that these risk factors would predict levels of somatic symptoms across the whole sample but did not make a prediction about whether the pattern of risk factors would differ between autistic and non-autistic people.

## Method

### Procedures

An online survey was conducted using Qualtrics. Universities across English-speaking countries (the United Kingdom, Ireland, the United States, Canada, Australia) were systematically listed, and support services were emailed with a request to distribute the link to students. Online support groups for ASCs and social media sites were also used. An information sheet was contained within the survey, and informed consent to participate was obtained by ticking the consent box before participants could complete the survey. At the end of the survey, debriefing information was provided along with an invitation to enter a prize draw to win a voucher. Measures were presented in the order outlined below.

### Participants

A total of 343 links to the survey were initiated: 3 participants declined to participate, 45 did not complete the consent page and an additional 92 gave consent but did not complete all of the study measures and were therefore excluded from the study. One participant was excluded for being unable to provide informed consent due to being below the age of 16 years. This left complete data on 202 participants (Table 1). Participants in the total sample were older adolescents and adults aged between 16 and 67 years (*M* = 31.33, *SD* = 12.90). They consisted of 146 females (72.3%), 48 males (23.8%), and 8 non-binary or transgender participants (4%). Fifty-one (25.2%) participants reported having a formal diagnosis of ASC. Thirty-two (15.8%) participants reported that they suspected they may be autistic. Another 119 (58.9%) participants reported that they did not have, nor suspect they had, an ASC. Participants self-reported their ethnicity in a free-text box, and the researchers later coded these answers into categories. No participants identified as Black; the remaining descriptions were coded as White/Caucasian, Asian or Mixed Race to reflect the terms used by participants. Where people reported their nationality rather than ethnicity, this was not coded.

**Table 1.** Characteristics of the sample.

	Diagnosed ASC	Suspected ASC	Non-ASC
Age, *M* (*SD*), range	33.9 (13.5), 16–62	36.1 (13.5), 19–67	28.9 (12.0), 17–64
Group comparison = *F*(2, 196) = 5.43, *p* = 0.005			
Gender, *n* (%)			
Male	17 (33.3%)	10 (31.3%)	21 (17.6%)
Female	29 (56.9%)	19 (59.4%)	98 (82.4%)
Non-binary/trans	5 (9.8%)	3 (9.4%)	0
Group comparison = χ^2^(4) = 20.16, *p* < 0.001			
Ethnicity, *n* (%)^a^			
White/Caucasian	44 (86.3%)	26 (81.3%)	79 (66.4%)
Mixed race	2 (3.9%)	1 (3.1%)	4 (3.4%)
Asian	0	0	6 (5%)
Group comparison = χ^2^(4) = 5.17, *p* = 0.270			
Education, *n* (%)			
No formal qualifications	2 (3.9%)	2 (6.3%)	1 (0.8%)
Secondary GCSE/equivalent	8 (15.7%)	3 (9.4%)	9 (7.6%)
Post-secondary	16 (31.4%)	5 (15.6%)	40 (33.6%)
Bachelor’s degree	14 (27.5%)	15 (46.9%)	46 (38.7%)
Postgraduate degree	9 (17.6%)	7 (21.9%)	18 (15.1%)
Other	2 (3.9%)	0	5 (4.2%)
Group comparison = χ^2^(10) = 12.90, *p* = 0.230			
Employment,^b^*n* (%)			
Full-time	14 (27.5%)	16 (50%)	49 (41.2%)
Part-time	9 (17.6%)	7 (21.9%)	29 (24.4%)
Unemployed (looking)	4 (7.8%)	1 (3.1%)	2 (1.7%)
Unemployed (not looking)	3 (5.9%)	1 (3.1%)	2 (1.7%)
Student	20 (39.2%)	5 (15.6%)	42 (35.3%)
Retired	1 (2.0%)	2 (6.3%)	2 (1.7%)
Self-employed	3 (5.9%)	2 (6.3%)	4 (3.4%)
Unable	5 (9.8%)	2 (6.3%)	1 (0.8%)

ASC: autism spectrum condition; GCSE: general certificate of secondary education; SD: standard deviation.

a Ethnicity data were missing for *n* = 40.

b Participants could select more than one of the employment options, so percentages do not sum to 100% and group comparisons could not be conducted.

Since participants who self-identify as autistic but do not have formal diagnoses are commonly included in autism research, we explored the most informative way to divide the groups. Fifty-one participants (17 male, 29 female, 5 non-binary) with formal diagnoses were assigned to the Diagnosed ASC group, 32 participants (10 male, 19 female, 3 non-binary) who suspected they were autistic were assigned to the Suspected ASC group, and the remaining 119 participants (21 male, 98 female) were assigned to the non-ASC group. To confirm the validity of these groupings, Autism Spectrum Quotient–Short Form (AQ-10) scores were compared, with the following means and standard deviations: Diagnosed ASC group, *M* = 7.86, *SD* = 1.41; Suspected ASC group, *M* = 5.56, *SD* = 2.68; Non-ASC group, *M* = 2.26; *SD* = 1.62. A one-way analysis of variance (ANOVA) confirmed that the group differences were significant, *F*(2, 199) = 187.30, *p* < 0.001, with post hoc comparisons showing that all group differences were significant (*p*s < 0.001). Therefore, given this difference on AQ-10 scores between the Diagnosed ASC and Suspected ASC groups, we decided not to combine them, and this three-way grouping was retained for subsequent analyses. The three groups differed significantly on age, with participants in the Suspected ASC group older than those in the No ASC group, and on gender, with a higher proportion of females in the No ASC group (Table 1).

### Materials

#### Demographics and health information

Participants answered questions concerning their age, gender, ethnicity, religion, education level and employment status. They were presented with a list of developmental disorders and mental health conditions and asked to tick if they had any of these conditions. They were then asked, ‘Do you have any serious physical health condition for which you are receiving treatment?’ with a free-text box for their answer. Finally, they were asked about their utilisation of health care services (based on Ritter et al., 2001). The survey questions are available in the supplementary material.

#### Autistic traits

The AQ-10 (Allison et al., 2012) is a non-diagnostic screening tool designed to indicate whether an adult may benefit from receiving a formal ASC assessment. The AQ-10 is scored on a scale from 1 to 10, with higher scores indicating higher levels of autistic traits and scores over six indicative of the need for a diagnostic assessment. Participants scored 10 statements on a 4-point Likert-type scale which ranged from *definitely agree* to *definitely disagree*. The statements included items such as ‘I often notice small sounds when others do not’. The AQ-10 provides a brief yet reliable and valid measure of autistic traits. Internal reliability was α = 0.83.

#### Alexithymia

The Toronto Alexithymia Scale (TAS-20; Bagby et al., 1994) is a tool designed to measure an individual’s level of alexithymia. The TAS-20 is scored on a scale from 20 to 100, with higher scores suggesting higher levels of alexithymia. Participants scored 20 statements on a 5-point Likert-type scale that ranged from *strongly disagree* to *strongly agree*. The statements included ‘I am often confused about what emotion I am feeling’. A revised scale using eight items from the TAS-20 has been found to be a more psychometrically robust measure of alexithymia in both autistic and non-autistic samples (Williams & Gotham, 2021), and therefore this General Alexithymia Factor Score–8 (GAFS-8) was utilised in the subsequent analyses. *T*-scores were generated using an online scoring tool (Williams, 2021). Internal reliability of the GAFS-8 was α = 0.91. The supplementary material presents additional comparisons between the GAFS-8 and TAS-20.

#### Interoception

The Body Awareness Questionnaire (BAQ; Shields et al., 1989) assesses an individual’s sensitivity to non-emotive bodily processes. The BAQ was scored on a scale of 18–126, with higher scores indicating higher levels of interoceptive sensibility. Participants scored 18 statements on a 7-point Likert-type scale that ranged from *not at all true of me* to *very true of me*. The items included statements such as ‘I notice differences in the way my body reacts to various foods’. The BAQ had high internal reliability, α = 0.89.

#### IU

The Intolerance of Uncertainty Scale–Short Form (IUS-12; Carleton et al., 2007) measures an individual’s ability to cope with uncertainty and ambiguity. The IUS-12 is scored on a scale of 12–60, with higher scores indicating higher levels of IU. Participants scored 12 statements on a 5-point Likert-type scale that ranged from *not at all characteristic of me* to *entirely characteristic of me*. The statements included items such as ‘Unforeseen events upset me greatly’. The IUS-12 had high internal reliability in the current study, α = 0.95.

#### Somatic symptoms

The Patient Health Questionnaire-15 (PHQ-15; Kroenke et al., 2002) measured levels of somatisation. The PHQ-15 was scored on a scale of 0–30, with higher scores indicating higher levels of somatic symptoms. The questionnaire lists 15 somatic symptoms, including stomach pain and back pain. Participants scored how much they had been affected by these problems over the past 4 weeks on a 3-point Likert-type scale that ranged from *not bothered at all* to *bothered a lot*. As Item 4 asks about menstrual symptoms, this item was dropped from the calculation of the total score. The PHQ-15 (with 14 remaining items) had an internal reliability of α = 0.84.

Abridged measures of theory of mind were included in the original study, but reliability was low, and the decision was taken not to include them in the analyses.

### Community involvement

The study was designed, conducted and written by the authors, one of whom is autistic (B.R.) and four of whom are not (F.L., M.H.-T, S.K. and S.D.). Data can be obtained from the Open Science Framework at https://t.ly/My8g.

### Data analysis

We first explored rates of physical health, health care utilisation and mental health in the three groups. We then ran group comparisons on the psychological variables of interest: somatic symptoms, alexithymia, IU and interoception. Finally, we used hierarchical regression to examine the influence of the psychological variables on somatic symptoms and to differentiate whether this influence differed by diagnostic group.

## Results

### Descriptive statistics

**Figure 1.**
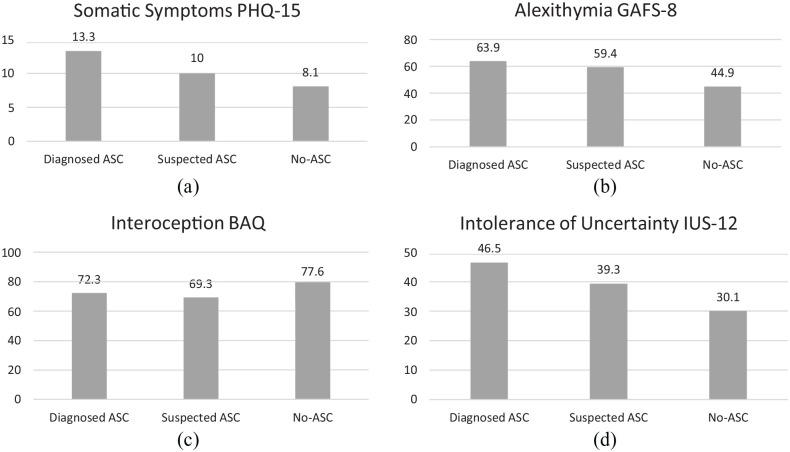
Comparison between the three groups on study variable mean scores. PHQ-15 = Patient Health Questionnaire-15; GAFS-8 = General Alexithymia Factor Score–8; BAQ = Body Awareness Questionnaire; IUS-12 = Intolerance of Uncertainty Scale–12.

Data were exported from Qualtrics into SPSS. Participants’ raw scores were scored according to the requirements of each measure. Table 2 and Figure 1 show the descriptive statistics for the study variables. Given the disparity between the diagnostic groups on gender and age, associations with the outcome variable were explored. Females showed higher levels of somatic symptoms than males, *t* = –2.31, *p* < 0.05, *d* = 0.38, consistent with previous research (Barsky et al., 2001), but not on the other variables of alexithymia, interoception or IU, *p*s > 0.464. Age was unrelated to any of the study variables, *p*s > 0.252. Gender and age were controlled for in subsequent analyses due to the group differences.

**Table 2.** Descriptive statistics for continuous study variables.

	Diagnosed ASC	Suspected ASC	No ASC
	*n* = 51	*n* = 32	*n* = 119
	*M* (*SD*), range	*M* (*SD*), range	*M* (*SD*), range
PHQ-15 Total Score	13.3 (5.8), 3–28	10 (5), 1–19	8.1 (4.6), 0–22
Mental Health	1.86 (1.34), 0–5	1.13 (1.21), 0–3	0.65 (.96), 0–4
AQ-10 Score	7.9 (1.4), 5–10	5.6 (2.7), 1–10	2.3 (1.6), 0–9
GAFS-8 T-Score	63.9 (9.36), 39.6–80.7	59.4 (11.7), 31.8–78	44.9 (10), 28.4–72.6
BAQ Total Score	72.3 (20.7), 36–126	69.3 (19.5), 37–108	77.6 (20.2), 24–119
IUS-12 Total Score	46.5 (9.1), 25–60	39.3 (12.2), 13–60	30.1 (10.2), 12–60
Outpatient visits	2.5 (2.6), 0–10	2.3 (2.6), 0–10	2.1 (3.2), 0–26

ASC: autism spectrum condition; SD: standard deviation; PHQ-15 = Patient Health Questionnaire-15; AQ-10 = Autism Quotient 10; GAFS-8 = General Alexithymia Factor; BAQ = Body Awareness Questionnaire; IUS-12 = Intolerance of Uncertainty Scale, Short Form.

### Health and health care utilisation

Thirty-one participants reported they had a serious physical health condition for which they were receiving treatment, including conditions such as cancer (*n* = 1), fibromyalgia (*n* = 3), chronic fatigue (*n* = 3) and asthma (*n* = 3). There was an association between ASC status and the presence/absence of diagnosed physical health conditions, χ^2^(2) = 6.86, *p* = 0.032 (Cramer’s *V* = 0.18, indicating a small to medium effect size), showing that the Diagnosed ASC participants were more likely to have a physical health condition (26% reported this) than the Non-ASC group (10% reported; 19% of the Suspected ASC group reported a physical health problem, which did not differ from the other two groups). Interestingly, despite the Diagnosed ASC group being more likely to report a health issue, rates for health care utilisation (frequency of GP or hospital outpatient appointments in the previous 6 months) were similar between the three groups, *F*(2, 198) = 0.28, *p* = 0.759, $\eta_{p}^{2}$ = 0.003. This may suggest under-utilisation of medical services by the Diagnosed ASC group. Autistic participants had higher rates of mental health conditions, *F*(2, 199) = 21.59, *p* < 0.001, $\eta_{p}^{2}$ = 0.18, and post hoc tests using Games–Howell showed the Diagnosed ASC group had higher rates of mental health conditions than the No ASC group and the Suspected ASC group, with no other significant comparisons.

### Group differences in outcome and psychological risk factors

An analysis of covariance (ANCOVA) was conducted to analyse group differences on the measure of somatic symptoms (PHQ-15), controlling for physical and mental health, age and gender. The assumption of homogeneity of variance was met. This test was significant, *F*(2, 187) = 6.67, *p* = 0.002, $\eta_{p}^{2}$ = 0.07, and post hoc tests showed that the No ASC group had significantly lower scores than the Diagnosed ASC group, with no other significant comparisons.

A series of ANCOVAs were conducted to investigate whether the groups differed on the psychological risk factors of interest: alexithymia, interoception and IU, controlling for age and gender. Levene’s test was non-significant for all variables, indicating equality of variances. There was no significant main effect of group on interoception (BAQ), *F*(2, 194) = 2.59, *p* = 0.078, $\eta_{p}^{2}$ = 0.03. There was a main effect of group on alexithymia (GAFS-8), *F*(2, 194) = 72.78, *p* < 0.001, $\eta_{p}^{2}$ = 0.43, with post hoc tests showing the No ASC group had significantly lower scores than the other two groups. There was a main effect of group on IU, *F*(2, 194) = 53.13, *p* < 0.001, $\eta_{p}^{2}$ = 0.35, with post hoc tests showing the No ASC group had lower scores than the other two groups, and the Suspected ASC group had lower scores than the Diagnosed ASC group.

### Predictors of somatic symptoms

We conducted a hierarchical regression to examine (a) whether somatic symptoms differed by ASC status and (b) whether the predictor variables (alexithymia, IU and interoception) predicted somatic symptoms. Zero-order correlations between the predictor and outcome variables were examined (Table 3). The Durbin–Watson test determined no independent errors within the data, and there was homoscedasticity among the data, therefore, assumptions for the regression were met.

**Table 3.** Zero-order correlations among dependent and independent variables.

	1	2	3^a^	4	5	6
1.PHQ Total Score						
2. Mental Health	.47**					
3. Physical Health^a^	.27**	.16*				
4.AQ-10 Score	.41**	.49**	.21**			
5.GAFS-8 T-Score	.50**	.49**	.14*	.68**		
6.BAQ Total Score	–.01	–.03	–.02	–.17*	–.12	
7. Intolerance of Uncertainty	.53**	.48**	.21**	.62**	.66**	–.05

ASC: autism spectrum condition; GAFS-8: General Alexithymia Factor Score–8; BAQ: Body Awareness Questionnaire.

a Point-serial correlations.

* *p* < 0.05; **p < 0.01.

In the first step of the model, we included autism status (see Table 4). This model was significant, indicating that autism diagnosis was a significant predictor of somatic symptoms, accounting for 17% of the variance. In the second step of the model, we added control variables: age, gender, mental and physical health. The second model was also significant and represented a significant *R*^2^ change, explaining 36% of the variance in somatic symptoms. Diagnosed autism, female gender, mental health and physical health were independent predictors. In the final step of the model, we added the psychological risk factors of interest, alexithymia (GAFS-8 score), IU (IUS-12 score) and interoception (BAQ score). The final model was also significant and represented a significant *R*^2^ change, explaining 45% of the variance in somatic symptoms. In the final model, autism diagnosis was no longer a significant predictor of somatic symptoms, while female gender, mental health, physical health, IU and alexithymia were independent predictors.

**Table 4.** Multiple hierarchical regression predicting PHQ-15 total score.

	*b*	*SE* B	β	*p*
Step 1				
Constant	8.05	0.46		0.000
Suspected ASC	1.88	1.04	0.12	0.072
Diagnosed ASC	5.21	0.85	0.42	0.000
	*R*^2^ = 0.17, *F*(2, 191) = 18.80, *p* < 0.001			
Step 2				
Constant	6.46	0.90		0.000
Suspected ASC	2.04	0.98	0.13	0.039
Diagnosed ASC	4.13	0.90	0.33	0.000
Mental Health	1.38	0.30	0.31	0.000
Physical Health	2.76	0.94	0.18	0.004
Age	–0.05	0.03	–0.12	0.056
Female gender	2.31	0.79	0.19	0.004
Other gender	–2.49	1.75	–0.09	0.157
	∆*R*^2^ = 0.20, *F*(7, 186) = 14.90, *p* < 0.001			
Step 3				
Constant	–1.38	2.14		0.521
ASC Suspected	–0.14	1.04	–0.01	0.895
ASC Diagnosed	1.39	1.01	0.11	0.170
Mental Health	0.82	0.30	0.19	0.007
Physical Health	2.44	0.89	0.16	0.007
Age	–0.036	0.03	–0.08	0.163
Female Gender	2.03	0.75	–0.17	0.008
Other Gender	–3.00	1.66	–0.11	0.073
Intolerance of Uncertainty	0.10	0.04	0.24	0.004
Alexithymia	0.09	0.04	0.23	0.009
Interoception	0.01	0.02	0.04	0.477
	∆*R*^2^ = 0.08, *F*(10, 183) = 14.44, *p* < 0.001			

PHQ-15: Patient Health Questionnaire-15; ASC: autism spectrum condition.

‘Other gender’ includes both non-binary and transgender participants.

According to these results, levels of somatic symptoms are explained by being female, by mental and physical health conditions, IU, and alexithymia, but not by autism itself. As autistic individuals are more likely to exhibit traits of IU and alexithymia, this explains the increased expression of somatic symptoms in ASC in comparison to the non-autistic participants.

## Discussion

This study investigated (a) whether somatic symptoms were higher in autistic than non-autistic individuals and (b) whether psychological factors of alexithymia, IU and interoceptive sensibility differed between autistic and non-autistic individuals, and whether those variables predicted levels of somatic symptoms. The findings of the study showed that (a) autistic participants had higher levels of somatic symptoms than those without a diagnosis (both the non-autistic and suspected autistic participants), (b) that alexithymia and IU differed by group, and that female gender, physical and mental health conditions, alexithymia and IU predicted levels of somatic symptoms, for autistic and non-autistic individuals. This is one of very few studies to compare rates of somatic symptoms between autistic and non-autistic people, and to examine psychological variables that predict these symptoms.

At the outset, it is important to note that autistic participants had higher rates of mental health conditions and were more likely to report a diagnosed physical health condition than non-autistic participants. This is consistent with previous research that shows a greater burden of physical illness (Mason et al., 2019) and mental illness (Lai et al., 2019) in autistic adults. In addition, our first finding confirmed the hypothesis that autistic people would experience higher levels of somatic symptoms. This finding is novel and important, as it suggests that autism is associated with more feelings of physical discomfort. This adds to the recent, emerging literature showing that autistic adults report high levels of pain, headache, dizziness and other somatic symptoms (Asztély et al., 2019; Lever & Geurts, 2016; Williams & Gotham, 2022). Somatic symptoms, irrespective of the medical cause, are associated with greater functional impairment and distress (Sharpe, 2013; Trimble, 2004). It is likely that stress and social conditions have an impact on physical health (Kirmayer et al., 2004), and given that autistic individuals are more vulnerable to psychosocial stressors (Griffiths et al., 2019), this may go some way to explaining these heightened physical symptoms. Recent work on central sensitisation in autism may also offer some promising developments in this field (Grant et al., 2022). This area deserves further attention in research and clinical settings, in order to understand the experience of somatic symptoms among autistic people and their impact on health care needs and quality of life.

Also consistent with previous research, we found that autistic people in the study had higher levels of alexithymia (Bird & Viding, 2014) and IU (Boulter et al., 2014; Neil et al., 2016). Contrary to predictions, we did not find differences between autistic and non-autistic participants on levels of interoceptive sensibility, with analyses showing a small effect size. As this measure has only been used in one other study of autistic adults (Fiene & Brownlow, 2015), further research is needed to understand this finding. Other paradigms that utilise experimental measures of interoception (e.g. heartbeat counting) may be employed in future research to test these associations, given that interoception may be a difficult construct to measure using self-report (e.g. Plans et al., 2020).

Finally, we found that levels of somatic symptoms were predicted by female gender, alexithymia, IU, and physical and mental health conditions, irrespective of ASC diagnosis. Women have consistently been found to report higher levels of somatic symptoms, potentially due to factors such as somatic sensitivity or socialisation (Barsky et al., 2001). Alexithymia has long been considered a negative prognostic factor for health outcomes (Kojima, 2012), as impaired subjective awareness and processing of emotions may mean that physical sensations associated with emotional arousal can be intensified and misinterpreted as illness symptoms, or can lead to impaired help-seeking, with negative impacts on physical health (Shalev, 2019; Taylor et al., 1997; Wise & Mann, 1995). The current findings support this association, though further research is required to understand the mechanisms linking alexithymia to somatic symptoms (Poquérusse et al., 2018). Exploring relations with neuroticism may be fruitful in this regard (see Matthews et al., 2009; Williams & Gotham, 2021).

Turning to IU, where individuals are high in IU, it may mean that the ambiguity associated with benign physical sensations may be more likely to draw the individual’s attention, and contribute to rumination or catastrophising (Carleton et al., 2007). In a similar vein, IU may lead an individual to be overly concerned about the unknown consequences associated with ambiguous social interactions, such as visiting a medical professional, and as a result, an individual may avoid seeking medical help, causing an increase in the severity of somatic symptoms (Kurita et al., 2013). This links with recently qualitative findings: Doherty et al. (2020) surveyed autistic adults who reported that difficulty in discerning when physical symptoms warrant medical attention is a barrier to attending general practice. Both alexithymia and IU are common factors within a range of psychopathology presentations; however, in the current study, they made an independent contribution to somatic symptoms over and above the number of mental health conditions the participants were experiencing.

We chose to include a subgroup of participants who reported that they suspected they may be autistic but did not have a formal diagnosis. Interesting patterns were found in the results: for most of the variables (Table 2, Figure 1), this subgroup scored midway between the Diagnosed ASC and No ASC group, which is unsurprising given that AQ-10 scores correlated highly with most of the predictor variables. On some of the variables, the Suspected ASC group aligned closely with the Diagnosed ASC group, namely, physical health problems and alexithymia. However, rates of IU were lower than the ASC group, and rates of mental health problems were no different to the non-autistic adults. One potential explanation for this is that the participants in this group may have been less likely to seek formal diagnosis due to the absence of additional mental health conditions, since mental health problems often motivate adults to seek autism assessment (Huang et al., 2020). Alternatively, they may not have sought formal diagnoses due to negative experiences with health care professionals or for other reasons (Lewis, 2016).

Interestingly, we did not find that autistic participants reported higher rates of health care utilisation than the groups without autism diagnoses, despite the fact that they were more likely to have diagnosed physical health conditions and higher levels of somatic symptoms. This may suggest that autistic participants in this study, who were largely young adults (mean age = 33), under-utilise medical services, which would be consistent with recent research (Burke & Stoddart, 2014; Calleja et al., 2020; Doherty et al., 2020; Mason et al., 2019). However, self-reports of health care usage are vulnerable to under-reporting (Ritter et al., 2001), so the current findings are tentative until replicated.

### Strengths and limitations

The main limitation of the current study was that participants were predominantly White and female, and a substantial minority had a third-level education. This means that the findings are not representative of the autistic population as a whole. Future research in this area should attempt to recruit a more racially diverse sample, through collaborations or targeted recruitment (see Jones & Mandell, 2020; Muthukrishna et al., 2020). Inclusion of measures of anxiety, depression and neuroticism would have enabled us to better control for their impact on somatic symptoms: these constructs tend to be closely related (Williams & Gotham, 2021), although the PHQ-15 has also been found to provide a reliable and valid assessment of somatisation as a distinct construct (Kroenke et al., 2010). Inclusion of a measure of quality of life would also have enabled a clearer picture of how somatic symptoms related to general well-being. Nevertheless, as autistic women are under-represented in research, this study provides valuable insights on this subgroup. The presence of an autistic researcher as part of the current study team was valuable in designing and interpreting the study in line with the priorities of the autism community (Fletcher-Watson et al., 2019).

### Clinical implications

Recent research has likely alerted clinicians practising in the area of ASCs to carefully screen for mental and physical health conditions during assessment and intervention (Lai et al., 2019; Matson & Goldin, 2013), which is also recommended by clinical guidance (National Institute for Health and Care Excellence (NICE), 2012). The findings of the current study make a further contribution in suggesting that autistic individuals’ somatic symptoms may be influenced by psychological factors which are common to ASC and may be particularly pertinent for autistic women. Somatic symptoms are associated with poor quality of life, mental ill health and reduced functioning (Henningsen et al., 2018), and thus may warrant attention in both physical and mental health settings. Within physical health settings, understanding the psychological factors which can influence individuals’ experience of somatic symptoms may help clinicians to more effectively address and alleviate concerns. Moreover, screening for psychological risk factors, such as alexithymia and IU, may enable mental health practitioners to gain a better understanding of clients’ distress. The findings also suggest the need to raise awareness of alexithymia and IU within the autism community to enable autistic people to choose interventions and support that may be of benefit to them. For example, psychological interventions that directly target alexithymia (e.g. Personalised Anxiety Treatment-Autism: Huntjens et al., 2020; Parr et al., 2020) or IU (e.g. Coping with Uncertainty in Everyday Situations: Rodgers et al., 2019) may have beneficial effects on the physical as well as mental health of autistic people if they wish to pursue therapeutic support.

## Supplemental Material

sj-docx-1-aut-10.1177_13623613221109717 – Supplemental material for Alexithymia and intolerance of uncertainty predict somatic symptoms in autistic and non-autistic adults Click here for additional data file. Supplemental material, sj-docx-1-aut-10.1177_13623613221109717 for Alexithymia and intolerance of uncertainty predict somatic symptoms in autistic and non-autistic adults by Fionnuala Larkin, Brianna Ralston, Sophie Jayne Dinsdale, Sakura Kimura and Marianna Emma Hayiou-Thomas in Autism

## Notes

1. While classified as a disorder in the *Diagnostic and Statistical Manual of Mental Disorders* (5th ed.; DSM-5), advocates within the autism community have voiced a preference for the term autism spectrum conditions (ASCs) which will be used throughout this article (see Bargiela et al., 2016; Kenny et al., 2016).
