# Supplementary material for: Alexithymia and intolerance of uncertainty predict somatic symptoms in autistic and non-autistic adults
Source: Autism. 2022 Jul 15;27(3):602–15. doi: 10.1177/13623613221109717 (PMC10076343; doi:10.1177/13623613221109717)
Supplement: sj-docx-1-aut-10.1177_13623613221109717 – Supplemental material for Alexithymia and intolerance of uncertainty predict somatic symptoms in autistic and non-autistic adults [file sj-docx-1-aut-10.1177_13623613221109717.docx]

Supplementary Material for Article: Alexithymia and Intolerance of Uncertainty Predict Somatic Symptoms in Autistic and Non-Autistic Adults

DEMOGRAPHICS QUESTIONS

Please try to complete the following questions in one sitting. However, if you can’t, you are able to come back to them at a later time providing you keep this tab open in your web browser.

With which gender do you identify?

- Male
- Female
- Other (please specify)
- Prefer not to say

What is your age (in years)?

Which ethnic group do you identify with?

Please specify any religious affiliation.

- Christian (including Church of England, Catholic, Protestant and all other Christian denominations)
- Buddhist
- Hindu
- Jewish
- Muslim
- Sikh
- Atheist
- None
- Other (please specify)

What is the highest level of education you have completed?

- Primary / secondary with no formal qualifications (e.g. no GCSE or school diplomas)
- Obtained GCSEs, high school degree or equivalent
- Post-secondary/ high school (e.g. A-levels, IB or BTec)
- Bachelor’s degree (e.g. BA, BSc)
- Postgraduate degree (e.g. MA, PhD)
- Other

What is your current employment status?

- Employed full-time (40+ hours a week)
- Employed part-time (less than 40 hours a week)
- Unemployed (currently looking for work)
- Unemployed (currently not looking for work)
- Student
- Retired
- Self-employed
- Unable to work

Do you have a formal diagnosis of ASD?

- Yes (please specify e.g. Asperger)
- No

If not, do you think you may have ASD?

- Yes
- No
- Maybe
- Is there any further information you would like to provide?

Do you have any other developmental disorders (e.g. ADHD) or mental health conditions (e.g. anxiety, depression, schizophrenia)? If so, please specify.

- Depression
- Anxiety
- Eating disorder, like Anorexia or Bulimia
- Substance or Alcohol Abuse
- Post-traumatic Stress Disorder (PTSD)
- Attention Deficit Hyperactivity Disorder (ADHD) or Attention Deficit Disorder (ADD)
- Oppositional Defiant Disorder or Conduct Disorder
- Learning Disability
- Specific Learning Difficulty
- Personality Disorder
- None
- Don’t know
- Other

Do you have any serious physical health conditions for which you are receiving treatment?

Have you visited any of these health professionals in the past 6-months?

- Internist
- General Practitioner (GP) or Family doctor
- Cardiologist
- Pulmonologist
- Neurologist
- Surgeon
- Rheumatologist
- Allergist
- Ophthalmologist
- Urologist
- Gynecologist
- Dermatologist
- None

How many times?

How many times have you visited the emergency room in the past 6-months?

How many times have you stayed in a hospital overnight in the past 6-months?

If there is anything else you think might be relevant, feel free to tell us.

**Information and Analyses for full TAS-20**

- Internal reliability of the TAS-20, α = .90. No ASC group (n = 119), α = .84. Suspected ASC group, α = .93. Diagnosed ASC group, α = .83.
- Internal reliability of the GAFS-8 T-score whole sample, α = .91. No ASC group (n = 119), α = .84. Suspected ASC group, α = .90. Diagnosed ASC group, α = .76.

Table S1

*Descriptive Statistics for TAS20*

*________________________________________________________________________*

Diagnosed ASC Suspected ASC No-ASC

n = 51 n = 32 n = 119

M (SD), Range M (SD), Range M (SD), Range

_________________________________________________________________________

TAS-20 Score 66.9(10.0), 43-84 59.8(14.4), 26-84 44.0(11.2), 24-74

GAFS-8 T-Score 63.9(9.4),39.6-80.7 59.4(11.7),31.8-78 44.9(10),28.4-72.6

___________________________________________________________________________

- Age did not correlate with **TAS-20**, *r* = .13, *p* = .078; or with **GAFS-8**, *r* = .08, *p* = .252.
- **TAS-20** mean scores did not differ between men and women, *t*(192) = 1.50, *p* = .134, *d* = 0.24; nor did **GAFS-8** mean scores, *t*(192) = .74, *p* = .464, *d* = 0.12.
- ANCOVA was run to investigate whether the groups (No ASC, Suspected ASC, Diagnosed ASC) differed on the alexithymia, controlling for age and gender. There was a significant main effect of group on **TAS-20** Total Score, *F*(2, 194) = 75.01, *p* < .001, η_p_^2^ = .44, with posthoc tests showing that all group comparisons were significant. There was a main effect of group on **GAFS-8**, *F*(2, 194) = 72.78, *p* < .001, η_p_^2^ = .43, with post-hoc tests showing the No ASC group had significantly lower scores than the other two groups.

Table S2

*Zero-order Correlations Amongst Dependent and Independent Variables, Including TAS-20*

1 2 3^a^ 4 5 6 7

1.PHQ Total Score

2.Mental Health .47**

3.Physical Health^a^ .27** .16*

4.AQ-10 Score .41** .49** .21**

5.GAFS-8 Score .50** .49** .14* .68**

6.BAQ Total Score -.01 -.03 -.02 -.17* -.12

7.Intolerance of Uncertainty .53** .48** .21** .62** .66** -.05

8.TAS-20 Score .49** .46** .15* .71** .94** -.16* .63**

*^a^ Point-serial correlations*

** *p* < .01, * *p* < .05

Table S3

*Multiple Hierarchical Regression Predicting PHQ Total Score with TAS-20*

b SE B β p

Step 1

Constant 8.05 .46 .000

Suspected ASC 1.88 1.04 .12 .072

Diagnosed ASC 5.21 .85 .42 .000

R^2^ = .17, *F*(2, 191) = 18.80, *p* < .001

Step 2

Constant 6.46 .90 .000

Suspected ASC 2.04 .98 .13 .039

Diagnosed ASC 4.13 .90 .33 .000

Mental Health 1.38 .30 .31 .000

Physical Health 2.76 .94 .18 .004

Age -0.05 .03 -.12 .056

Female gender 2.31 .79 .19 .004

Other gender -2.49 1.75 -.09 .157

∆R^2^ = .20, *F*(7, 186) = 14.90, *p* < .001

Step 3

Constant -1.39 2.10 .501

ASC Suspected -0.11 1.02 -.01 .913

ASC Diagnosed 1.23 1.01 .10 .228

Mental Health 0.82 .30 .18 .006

Physical Health 2.43 .89 .16 .007

Age -.04 .03 -.10 .106

Female Gender 2.20 .75 -.18 .004

Other Gender -2.94 1.65 -.11 .076

Intol. of Uncertainty 0.10 .03 .23 .004

Alexithymia (TAS20) 0.09 .03 .25 .003

Interoception 0.01 .02 .05 .355

∆R^2^ = .08, *F*(10, 183) = 14.78, *p* < .001
